# Enhancing decision-making strategies in treatment for unruptured intracranial aneurysms: a novel analytical approach using PHASES, ELAPSS and UIATS scores for microsurgical clipping outcome prediction

**DOI:** 10.1007/s10143-025-03683-y

**Published:** 2025-07-11

**Authors:** Philipp A. Geiger, Christian Preuss-Hernandez, Nikolaus Kögl, Jeannine Rey, Claudius Thomé, Ondra Petr

**Affiliations:** 1 Department of Neurosurgery, Medical University Innsbruck, Tyrol, Austria; 2 Department of Neurosurgery & Neurooncology, First Faculty of Medicine, Charles University, Prague, Czech Republic

**Keywords:** Unruptured intracranial aneurysms, Surgical outcome, PHASES score, ELAPSS score, UIATS score, Predictive analysis

## Abstract

*Objective*: The management of unruptured intracranial aneurysms (UIA) is complex, balancing the risks of surgical intervention against aneurysm rupture. The PHASES, ELAPSS, and UIATS scoring systems have been developed to assist in clinical decision-making, but their efficacy in predicting surgical outcome remains unclear. *Methods*: In this monocentric, retrospective, observational study, we included 380 patients with UIA from January 2010 to January 2021. We assessed the predictive value of the PHASES, ELAPSS, and UIATS scores in determining clinical outcome post-surgery, including different variables. Statistical analyses, including Principal Component Analysis and Multiple logistic and linear regression, were employed to analyze the data. *Results*: Our cohort of 380 predominantly female patients (71.3%) had a mean age of 54.7 years. The PHASES and UIATS pro-conservative scores were significant predictors of poor clinical outcome (*p* = 0.03 and *p* = 0.04, respectively), while the ELAPSS score was predictive of new neurological deficits post-surgery (*p* = 0.01). Aneurysm size was significantly associated with new neurological deficits but not with long-term clinical performance/outcome. *Conclusions*: The study underscores the utility of PHASES, ELAPSS, and UIATS scores in preoperative risk stratifications. Conservative PHASES and UIATS scores were associated with poor outcome, therefore supporting their predictive value of non-operative management. Our findings suggest the routine implementation of these scores into clinical practice could improve the management of UIAs.

## Introduction

Managing unruptured intracranial aneurysms (UIAs) presents a complex challenge especially due to the intact neurological conditions in vast majority of these patients. It involves weighing the inherent risks of surgical intervention against the potentially catastrophic consequences of aneurysm rupture [1]. Advancements in neurosurgical techniques and endovascular interventions in recent years have broadened the therapeutic options for brain aneurysm treatment [2]. The decision-making process for treatment—be it intervention, observation, or surgical—requires comprehensive assessment. This includes considering the aneurysm’s size, location, morphology, morphologic changes over time as well as the patient’s health status and life expectancy [3]. 

Several risk assessment scores, such as PHASES, ELAPSS, and UIATS, have been developed to assist clinicians in this complex decision-making process. These scoring systems provide quantitative evaluations of rupture risk, growth risk, and the benefit-risk ratio of intervention versus conservative management [4]. However, their effectiveness in predicting clinical outcome post-surgery remains uncertain.

The PHASES score assesses cumulative rupture risk based on 6 parameters: Population, hypertension, age, size of the aneurysm, earlier SAH from another aneurysm, and site of the aneurysm [5]. The ELAPSS score, on the other hand, estimates the likelihood of aneurysm growth, factoring in earlier SAH, aneurysm location, age above 60 years, population, aneurysm size, and shape [6]. The UIATS (Unruptured Intracranial Aneurysm Treatment Score) integrates patient-specific and aneurysm-specific factors, guiding treatment decisions [7]. 

While these scores offer structured decision-making approaches, they have certain limitations. Real-world clinical scenarios often present nuances that standardized scoring systems may not fully capture. Moreover, these scores focus on pre-surgical situations and do not address postoperative morbidity risks if surgical intervention is chosen.

Given the comprehensive nature of these risk scores, encompassing various patient and aneurysm-specific factors, they might be extendable beyond their current applications. They could potentially predict post-operative outcome and the likelihood of neurological deficits following aneurysm intervention. If validated, this extension could enhance patient counseling and surgical planning, providing a more holistic understanding of the risks involved.

## Methods

We conducted a monocentric, retrospective, observational study at an academic tertiary care center of surgically treated patients harboring unruptured intracranial aneurysms. All patients with intracranial aneurysms, irrespective of rupture status, are evaluated by an interdisciplinary cerebrovascular board - comprising neurosurgeons, neuroradiologists, and neurologists - in accordance with institutional standards at our university hospital.

Patients eligible for the study presented with a diagnosed UIA opting for surgical treatment. Patients under the age of 18 or older than 85 years were excluded from the study. Pregnancy and significant morbitiy with an estimated life expectancy of ≤ 2 years were additional exclusion factors. Importantly, patients were not respected for this study in case of conservative management or the decision to proceed with endovascular treatment.

Demographics included gender and age, while the medical condition was assessed using the American Society of Anesthesiologists (ASA) score. Any anticoagulation therapy was noted.

The number of aneurysms per patient and their location was noted. Each aneurysm was assessed for morphology, size, contrast enhancement and intraluminal thrombosis. Giant aneurysms were classified as equal or larger than 25 mm in size. Screening 644 patients with unruptured intracranial aneurysms, 264 cases were excluded. At the time of enrollment PHASES, ELAPSS, and UIATS were deteremined. Outcome measures included the Modified Rankin Scale (mRS) at admission and discharge, as well as any new neurological deficits at discharge. The study period ranged from January 2010 to January 2021, with all patients treated at the Departments of Neurosurgery and Neuroradiology of the Medical University Innsbruck.

Intracranial aneurysm treatments were performed via microsurgical clipping, adhering to institutional standard operating procedures. Depending on aneurysm localization, surgical approaches included pterional, temporal, or suboccipital craniotomy. Intraoperative clip occlusion was confirmed using indocyanine green (ICG) angiography, and postoperative digital subtraction angiography (DSA) was routinely conducted during the hospital stay as part of our standard care protocol. Patient data were collected using the KIS PowerChart (Oracle Cerner, Austin, Texas) patient data information system and Excel^®^.

The primary endpoint was the predictability of unfavourable clinical outcome defined as mRS > 2. Secondary endpoints included the presence of new neurological deficits post-incident and patient and aneurysm-associated risk factors for unfavourable outcome. Statistical analysis was conducted using JMP Pro (Version 17, SAS Institute Inc.), employing Principal Component Analysis and multiple logistic and linear regression for retrospective data analysis. We focused on two dependent variables: unfavorable outcome at discharge (defined by mRS 3–6) and new neurological deficits at discharge. Normality was tested via Shapiro-Wilk and Anderson-Darling tests. As data were not normally distributed– even after excluding outliers -, we used the Mann-Whitney U test for single factors and multiple logistic regression to account for confounding factors.

Seven cases were excluded for the analysis of location because of incomplete documentation.

## Results

### Patient sample and demographics

Our study respected 644 interdisciplinary reviewed cases, of which 380 patients underwent surgical treatment for UIA. These 380 patients were considered as study cohort comprising 271 females (71.3%) with an average age of 54.7 ± 10.8 years. Anticoagulation therapy was administered in 107 patients (28.2%). The cohort’s average American Society of Anesthesiologists (ASA) score was 2.05 (SD 0.56), indicating a moderate anesthetic risk profile.

### Aneurysm characteristics

The size of the aneurysms varied from 1.5 to 50 mm, with an average of 8.03 mm (SD 5.28). There were 7 giant aneurysms with a size of 25 mm and larger.

On average, patients had 2.03 aneurysms each (SD 1.4). The majority of aneurysms were saccular (353 cases, 95.7%), with 15 fusiform aneurysms (4.1%). Intraluminal thrombosis was observed in 48 patients (12.6%). Aneurysm size showed statistical significance for new neurological deficits (*p* = 0.01, OR 1.06) but not for clinical performance status (*p* = 0.22). Neither intraluminal thrombosis nor the number of aneurysms per patient showed statistical significance (*p* = 0.27, *p* = 0.09, respectively).

**Fig. 1:**
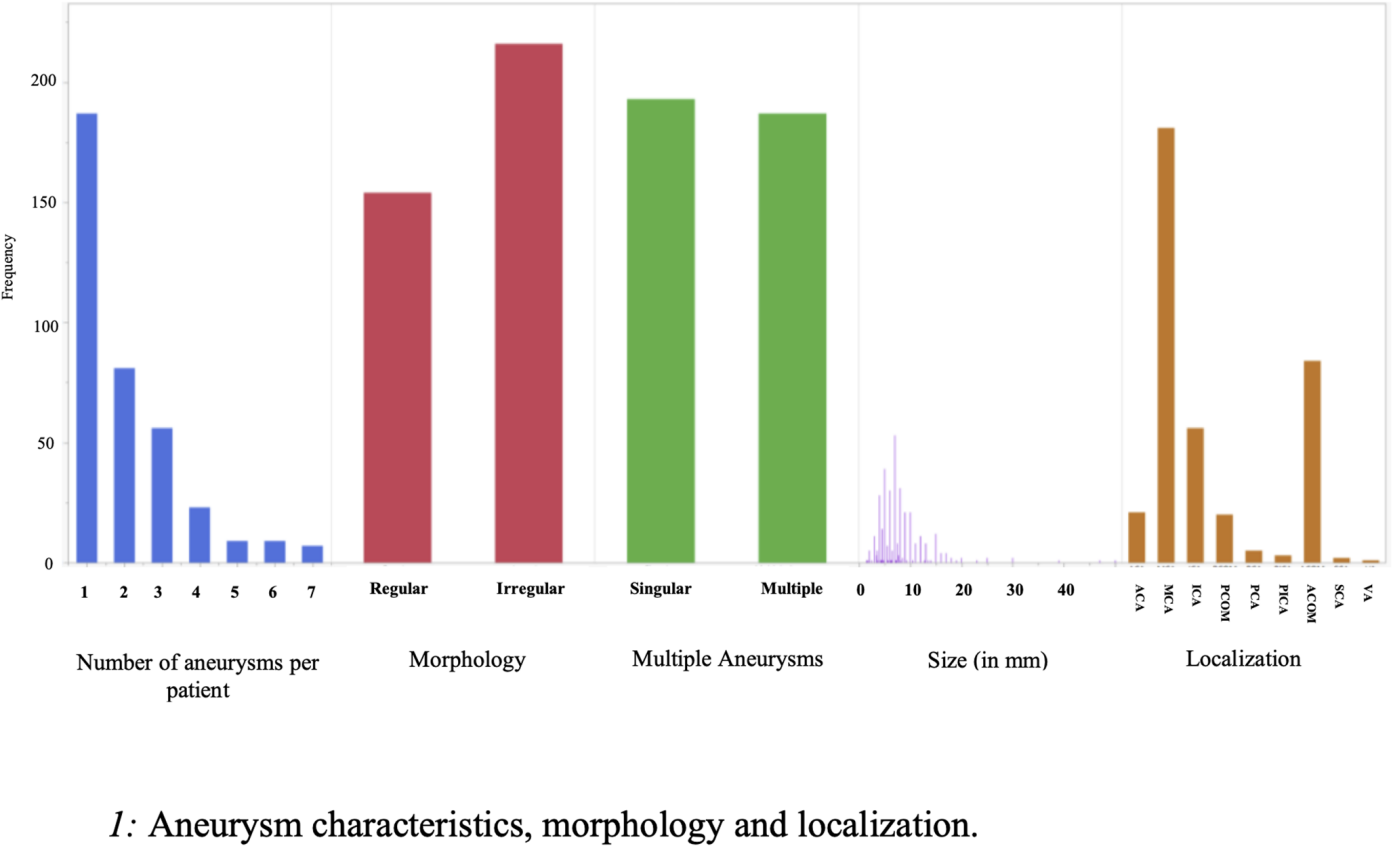
Aneurysm characteristics, morphology and localization

**Fig. 2:**
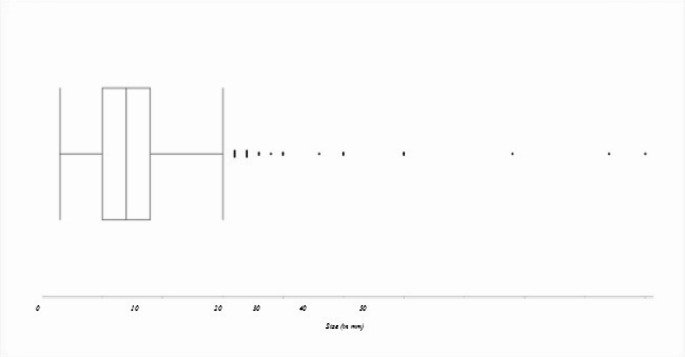
Box-plot for aneurysm sizes with visualization of outliers. X-axis represents size in mm

The aneurysm characteristics are listed in Table 1; Figs. 1 and 2.

**Table 1:** Aneurysm characteristics and localizations. Abbrv.: aca = anterior cerebral artery, mca = middle cerebral artery, ica = internal carotid artery, pcom = posterior communicating artery, pca = posterior cerebral artery, pica = posterior inferior cerebellar artery, acom = anterior communicating artery, sca = superior cerebellar artery, va = vertebral artery

LOCALIZATION		*N*	%
ACA		21	5.6
MCA		181	48.5
ICA		56	15
PCOM		20	5.4
PCA		5	1.3
PICA		3	0.8
ACOM		84	22.5
SCA		2	0.54
VA		1	0.27
Total		373	100
Missing		7	
**MORPHOLOGY**			
Regular		154	41,6
Irregular		216	58,4
		370	10 missing (due to missing records)
**SIZE**			
< 25 mm		373	98.2
≥ 25 mm		7	1.8
Mean		8 (5,3)	
Median		7	
Mean without giant aneurysms		7.5 (3.6)	
Median without giant aneurysms		7	
			0 missing
**MULTIPLE ANEURYSMS**			
Singular	193	193	50.8
Multiple	187	187	49.2
			0 missing

### Scoring systems

**Fig. 3:**
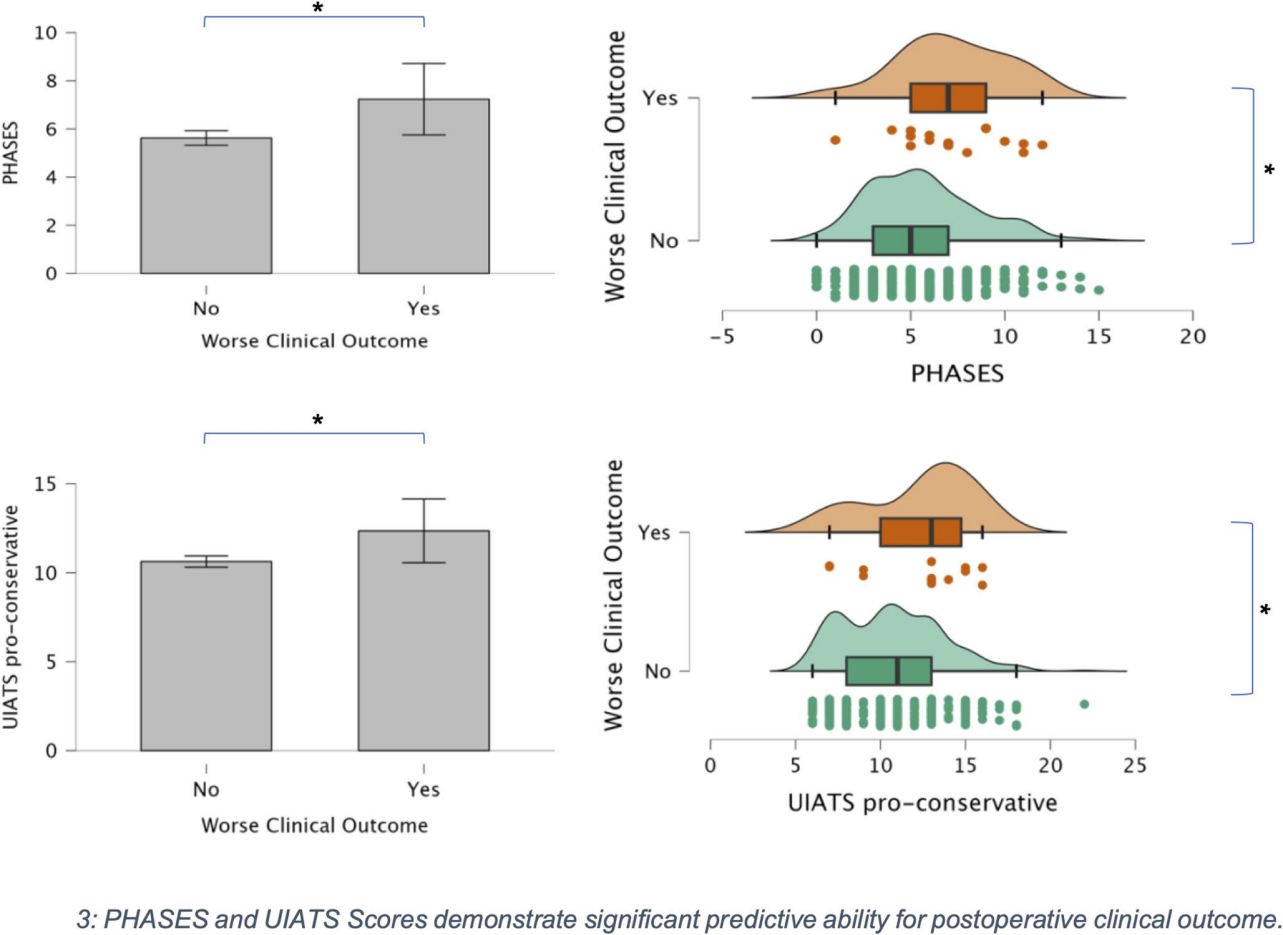
PHASES and UIATS Scores demonstrate significant predictive ability for postoperative clinical outcome

**Fig. 4:**
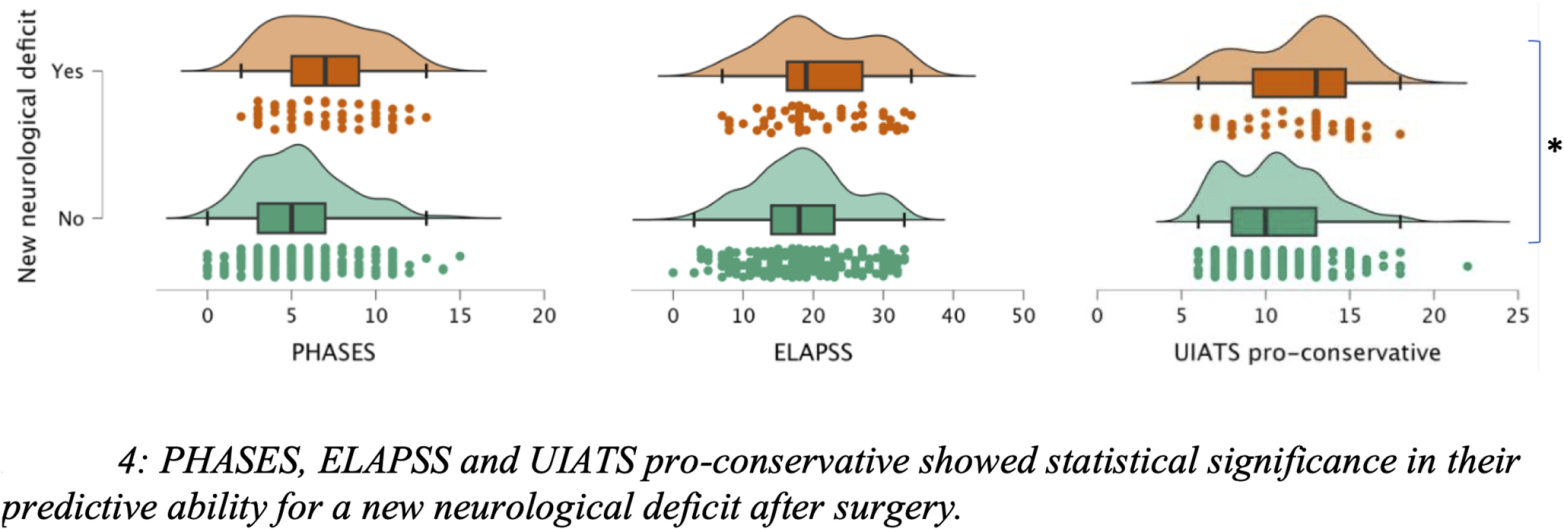
PHASES, ELAPSS and UIATS pro-conservative showed statistical significance in their predictive ability for a new neurological deficit after surgery

The average PHASES score was 5.7 (SD 2.9), ranging from 0 to 15 points. The ELAPSS score averaged at 18.7 (SD 7.1), with a range from 0 to 34 points. The UIATS pro-repair score averaged 13.3 points (SD 4.4), ranging from 4 to 31 points, while the pro-conservative score averaged 10.7 (SD 3), with a range from 6 to 22 points. In principal component analysis, the PHASES, ELAPSS, and UIATS pro-conservative scores demonstrated significant predictive value for clinical outcome (*p* = 0.04, 0.02, and 0.006, respectively). Multiple logistic regression confirmed the predictive capacity of PHASES (*p* = 0.03, OR 1.193) and UIATS pro-conservative (*p* = 0.04, OR 1.202) for unfavorable clinical outcome. ELAPSS did not show significance for mRS at discharge (*p* = 0.16). In terms of new neurological deficits, PHASES (*p* = 0.001, OR 1.173), ELAPSS (*p* = 0.01, OR 1.054), and UIATS pro-conservative (*p* = 0.003, OR 1.171) were significantly linked to unfavorable outcome *(see* Figs. 3 and 4). Table 2 summarizes data findings according to the score-associated factors.

**Table 2:** Score-related risk factors of our patient cohort

	Overall (*N* = 380)
**Age**	
Mean (SD)	54.7 (10.8)
Range	20.7–82.0
**Sex**	
Male	109 (28.7%)
Female	271 (71.3%)
**Arterial Hypertension**	
No	226 (59.5%)
Yes	154 (40.5%)
**Aneurysm Size**	
< 7 mm	162 (44%)
7–9.9 mm	122 (33%)
10–19.9 mm	75 (20%)
≥ 20 mm	10 (2.7%)
**Prior SAH**	
No	209 (82.9%)
Yes	64 (17.2%)
**Nicotine**	
No	234 (61.6%)
Yes	146 (38.4%)
**Positive family history**	
No	303 (82.6%)
Yes	64 (17.4%)
**PHASES Score**	
Mean (SD)	5.7 (2.9)
Range	0–15
**ELAPSS Score**	
Mean (SD)	18.7 (7.1)
Range	0–34
**UIATS pro-repair**	
Mean (SD)	13.3 (4.4)
Range	4–31
**UIATS pro-conservative**	
Mean (SD)	10.7 (3.0)
Range	6–22

### Patient characteristics

Higher age was statistically significant for new neurological deficits post-surgery (*p* = 0.02, OR 1.037) and for worse clinical outcome at discharge (*p* = 0.01, OR 1.063). Sex and ASA score showed no statistical significance (*p* = 0.14, *p* = 0.25, respectively).

## Discussion

Our cohort of 380 subjects, predominantly females (71.3%), with an average age of 54.7 years, reflects the known demographic distribution of unruptured intracranial aneurysms. The anticoagulation usage in approximately one third of patients and the average ASA score underscore a moderate anesthetic risk profile. The range of aneurysm sizes and the predominance of saccular types align with typical clinical presentations.

Pro-conservative PHASES and UIATS scores were found to be associated with poor clinical outcome following surgical intervention, while higher ELAPSS scores were linked to new neurologic deficits. Our findings suggest that these scores may predict clinical outcome and could be used for the perioperative risk stratification for e.g. the occurrence of new neurological deficits.

Our results resonate with the study by James Feghali et al. [8], which examined the correlation between these scores and clinical practices at referral centers for managing unruptured intracranial aneurysms [8]. Our findings build upon their research, highlighting the potential extension of these scores’ application.

The scoring systems demonstrated relevant predictive value in our principal component analysis for clinical outcome. PHASES and UIATS pro-conservative scores were notably predictive of unfavorable clinical outcome, as confirmed by multiple logistic regression. This finding is particularly important as the indication for surgery is generally a shared decision making process. Pro-conservative PHASES and UIATS scores (as originally intended) not only support a watch-and-wait policy, but now have been proven to be predictive for poor clinical outcome. This can be partially explained by the factors included in these scoring systems such as age, which was associated with new neurological deficits and worse clinical outcome at discharge. This finding suggests that its relevance is underscored within the above mentioned scoring systems. Furthermore, frailty remains unrespected and need to be confirmed by future studies.

While ELAPSS showed predictive ability for immediate postoperative complications (new neurological deficits), it did not predict long-term clinical outcome. The key distinction between the PHASES and ELAPSS scores lies in the inclusion of aneurysm morphology in the latter, which may influence the prediction of short-term outcomes. Conversely, this suggests that irregular morphology may not significantly impact the long-term prediction of clinical outcomes.

The study by Qingyuan Liu et al. [4] provides additional context for the predictive value of these scores in preoperative assessments, complementing our findings [9]. 

The potential of integrating machine learning-based predictive analytics with traditional scoring systems, as explored in the pilot study by V. Staartjes et al., is an exciting development [4]. It suggests a future where advanced analytics could augment the predictive accuracy of established scores like PHASES, ELAPSS, and UIATS.

Our findings are further supported by a comprehensive review by Algra et al. [10], which corroborated several risk factors encompassed in the PHASES and ELAPSS scores. For neurosurgical treatment, the pooled rate of clinical complications was 8.34% (95% CI, 6.25–11.10%), while the case-fatality rate remained substantially lower at 0.10% (95% CI, 0.00–0.20%). Risk factors for neurosurgical complications included age (OR per year increase: 1.02), coagulopathy (OR: 2.14), anticoagulation use (OR: 6.36), smoking (OR: 1.95), hypertension (OR: 1.45), diabetes (OR: 2.38), congestive heart failure (OR: 2.71), posterior circulation aneurysms (OR: 7.25), and aneurysm calcification (OR: 2.89). It emphasized the need for standardized definitions of complications and better reporting practices to improve risk estimation accuracy [10]. 

Our study also highlights the practicality of these scoring systems in clinical decision-making, as analyzed by Sandro Hügli et al. [11] in their examination of real-world decision-making versus score recommendations [11]. The significant association of aneurysm size with immediate postoperative morbidity, but not with long-term outcome, adds an important dimension to surgical planning, as some patients seem to recover from temporary worsening. The absence of increased morbidity with intraluminal thrombosis and multiple aneurysms per patient is noteworthy.

### Limitations

The limitations of our study primarily stem from its retrospective design, which inherently restricts the ability to establish causal relationships and may introduce selection bias. Furthermore, the absence of a control group limits the robustness of comparisons and generalizability of the findings. Irrespective of preoperative scores, the decision to opt for surgery remains a combination of surgeon and patient preferences based on a shared decision-making process. This variability could potentially influence patient care and clinical outcomes due to differences in individual experience and technique. These factors should be considered when interpreting the results, and future prospective studies with standardized protocols and inclusion of control groups are warranted to validate our findings and address these limitations comprehensively. Lastly, giant aneurysms played a subordinate role, with little effect on the overall results.

## Conclusions

Our findings, corroborated by current literature, emphasize and validate the significance of PHASES, ELAPSS, and UIATS scores in preoperative assessment and surgical risk evaluation for unruptured intracranial aneurysms. Pro-conservative scores were associated with an increased risk of unfavorable neurological outcomes following microsurgical clipping. These scoring systems offer valuable predictive insights for clinical decision-making, potentially improving patient outcome and guiding surgical planning. Future studies should aim to explore the integration of these scores with upcoming artificial intelligence and evaluate their applicability across a more heterogenous patient populations to ensure that their benefits are universally accessible.

## Data Availability

No datasets were generated or analysed during the current study.
